# Supplementary material for: Intake of Red and Processed Meat, Use of Non-Steroid Anti-Inflammatory Drugs, Genetic Variants and Risk of Colorectal Cancer: A Prospective Study of the Danish “Diet, Cancer and Health” Cohort
Source: Int J Mol Sci. 2019 Mar 5;20(5):1121. doi: 10.3390/ijms20051121 (PMC6429260; doi:10.3390/ijms20051121)
Supplement: Supplementary file 1 [file ijms-20-01121-s001.pdf]

Supplemental Table 1. Tertile analyses of polymorphisms and dietary factors

	1. tertile	2.tertile	3. tertile	1. tertile	2. tertile	3. tertile	P-value[1]	1. tertile	2.tertile	3. tertile	1. tertile	2. tertile	3. tertile	P-value[1]
	Nc/Ns	Nc/Ns	Nc/Ns	IRR (95%CI)	IRR (95%CI)	IRR (95%CI)		Nc/Ns	Nc/Ns	Nc/Ns	IRR (95%CI)	IRR (95%CI)	IRR (95%CI)	
SLC25A20 rs7623023	Red and processed meat							Fibre						
AA	92/240	144/218	125/227	1.00 (ref)	1.87(1.19-2.93)	1.39(0.85-2.29)		125/222	131/229	105/234	1.00 (ref)	1.03(0.65-1.64)	0.87(0.48-1.56)	
GA+GG	176/347	181/345	199/345	1.38(0.91-2.07)	1.44(0.94-2.19)	1.59(1.00-2.54)	0.03	199/339	190/330	167/368	1.05(0.71-1.54)	1.09(0.71-1.67)	0.92(0.54-1.57)	1.00
	Fruit and vegetables							Alcohol						
AA	129/224	127/220	105/241	1.00 (ref)	1.16(0.74-1.82)	0.88(0.53-1.46)		113/225	120/239	128/221	1.00 (ref)	0.90(0.58-1.38)	0.99(0.62-1.57)	
GA+GG	197/332	191/353	168/352	1.06(0.73-1.56)	1.05(0.70-1.56)	1.07(0.65-1.75)	0.38	193/347	172/343	191/347	1.06(0.71-1.57)	0.93(0.62-1.39)	1.03(0.68-1.57)	1.00
PRKAB1 rs4213	Red and processed meat							Fibre						
TT+TG	239/537	301/503	291/526	1.00 (ref)	1.40(1.02-1.90)	1.22(0.84-1.76)		284/513	301/507	246/546	1.00 (ref)	1.12(0.81-1.56)	0.90(0.57-1.44)	
GG	29/ 50	25/ 57	34/ 45	1.52(0.77-2.98)	1.03(0.52-2.04)	1.75(0.89-3.43)	0.09	40/ 47	21/ 49	27/ 56	1.85(1.01-3.41)	0.81(0.38-1.73)	0.97(0.44-2.15)	0.04
	Fruit and vegetables							Alcohol						
TT+TG	292/514	290/519	249/533	1.00 (ref)	1.10(0.81-1.49)	1.02(0.68-1.53)		274/511	266/525	291/530	1.00 (ref)	0.89(0.66-1.20)	0.96(0.69-1.33)	
GG	34/ 42	29/ 50	25/ 60	1.74(0.91-3.34)	1.23(0.62-2.43)	0.83(0.39-1.74)	0.11	31/ 61	29/ 56	28/ 35	1.06(0.57-1.97)	1.01(0.52-1.97)	1.33(0.64-2.77)	0.76
LPCAT1 rs7737692	Red and processed meat							Fibre						
AA+GA	227/517	280/502	290/486	1.00 (ref)	1.32(0.96-1.81)	1.36(0.93-2.00)		281/495	272/487	244/523	1.00 (ref)	1.00(0.72-1.40)	0.90(0.57-1.43)	
GG	40/ 66	46/ 58	34/ 86	1.49(0.82-2.72)	2.20(1.20-4.06)	0.95(0.51-1.77)	0.02	43/ 67	49/ 70	28/ 73	1.25(0.71-2.20)	1.42(0.80-2.53)	0.74(0.35-1.57)	0.22
	Fruit and vegetables							Alcohol						
AA+GA	290/484	265/499	242/522	1.00 (ref)	0.98(0.71-1.33)	0.92(0.62-1.39)		258/493	262/512	277/500	1.00 (ref)	0.91(0.67-1.23)	1.00(0.71-1.39)	
GG	36/ 72	54/ 72	30/ 66	0.91(0.50-1.63)	1.51(0.87-2.62)	0.99(0.48-2.02)	0.19	47/ 74	32/ 69	41/ 67	1.32(0.75-2.33)	0.99(0.53-1.85)	1.07(0.58-1.96)	0.77
PLA2G4A rs4402086	Red and processed meat							Fibre						
AA+GA	247/534	289/522	288/526	1.00 (ref)	1.25(0.92-1.70)	1.21(0.84-1.75)		293/512	289/517	242/553	1.00 (ref)	1.04(0.75-1.45)	0.88(0.55-1.40)	
GG	19/ 52	31/ 40	35/ 45	0.84(0.38-1.82)	1.85(0.93-3.67)	1.67(0.84-3.31)	0.31	26/ 49	31/ 41	28/ 47	1.08(0.55-2.11)	1.36(0.66-2.78)	1.14(0.53-2.46)	0.84
	Fruit and vegetables							Alcohol						
AA+GA	293/509	289/525	242/548	1.00 (ref)	1.05(0.77-1.43)	0.92(0.61-1.38)		278/534	263/527	283/521	1.00 (ref)	0.91(0.68-1.23)	0.99(0.72-1.38)	
GG	28/ 46	28/ 47	29/ 44	1.13(0.58-2.20)	1.27(0.64-2.51)	1.24(0.59-2.63)	0.89	25/ 38	27/ 53	33/ 46	1.36(0.66-2.82)	0.95(0.47-1.90)	1.33(0.68-2.58)	0.73
ALOX5 rs3780894	Red and processed meat							Fibre						
AA+GA	256/568	317/551	314/566	1.00 (ref)	1.35(1.00- 1.82)	1.26(0.87- 1.81)		310/546	312/547	265/592	1.00 (ref)	1.04(0.76-1.44)	0.88(0.56-1.39)	
GG	9/ 19	7/ 12	8/ 7	1.15(0.39- 3.45)	1.34(0.36- 5.00)	2.43(0.50-11.93)	0.68	12/ 15	7/ 13	5/ 10	1.35(0.47-3.90)	1.21(0.30-4.82)	1.06(0.22-5.05)	0.97
	fruit and vegetables							alcohol						
AA+GA	314/541	302/563	271/581	1.00 (ref)	1.01(0.75- 1.37)	0.96(0.65- 1.44)		293/557	284/569	310/559	1.00 (ref)	0.89(0.67-1.19)	0.99(0.72-1.37)	
GG	9/ 17	13/ 10	2/ 11	0.87(0.28- 2.72)	3.14(0.96-10.26)	0.42(0.05- 3.34)	0.04	11/ 14	7/ 13	6/ 11	1.53(0.49-4.76)	1.25(0.38-4.15)	0.80(0.18-3.51)	0.64
PTGER3 rs6685546	red and processed meat							fibre						
TT+TC	261/574	316/547	313/550	1.00 (ref)	1.32(0.98-1.78)	1.24(0.86-1.78)		316/544	310/544	264/583	1.00 (ref)	1.01(0.73-1.39)	0.86(0.54-1.35)	
CC	7/ 11	8/ 15	12/ 21	1.20(0.31-4.58)	1.02(0.30-3.44)	1.11(0.38-3.21)	0.82	8/ 13	11/ 14	8/ 20	0.70(0.20-2.43)	1.41(0.46-4.34)	0.68(0.20-2.31)	0.49
	Fruit and vegetables							Alcohol						
TT+TC	319/536	306/556	265/579	1.00 (ref)	1.01(0.75-1.37)	0.92(0.62-1.37)		297/563	282/555	311/553	1.00 (ref)	0.91(0.68-1.21)	0.99(0.72-1.37)	
CC	8/ 18	12/ 14	7/ 15	0.58(0.18-1.89)	1.52(0.50-4.59)	0.86(0.23-3.14)	0.31	6/ 9	12/ 24	9/ 14	0.99(0.23-4.27)	0.81(0.30-2.19)	0.90(0.27-3.04)	0.99
TP53 rs1042522	Red and processed meat							Fibre						

GG	154/296	181/328	168/316	1.00 (ref)	1.08(0.74-1.59)	1.02(0.66-1.57)		179/298	174/310	150/332	1.00 (ref)	0.97(0.65-1.43)	0.84(0.51-1.39)	
GC+CC	111/291	143/233	154/253	0.72(0.48-1.07)	1.24(0.83-1.86)	1.18(0.75-1.85)	0.04	142/260	146/247	120/270	0.89(0.61-1.30)	1.05(0.69-1.58)	0.86(0.50-1.46)	0.65
	Fruit and vegetables							Alcohol						
GG	172/303	175/320	156/317	1.00 (ref)	1.06(0.73-1.54)	1.02(0.64-1.62)		172/304	154/319	177/317	1.00 (ref)	0.78(0.54-1.13)	0.89(0.60-1.31)	
GC+CC	151/248	142/251	115/278	1.07(0.73-1.56)	1.09(0.73-1.63)	0.87(0.55-1.39)	0.54	131/268	137/260	140/249	0.81(0.54-1.20)	0.87(0.59-1.29)	0.94(0.62-1.41)	0.27
CCAT2 rs6983267	Red and processed meat							Fibre						
GG	87/168	101/151	118/151	1.00 (ref)	1.25(0.76-2.05)	1.46(0.87-2.46)		109/157	106/146	91/167	1.00 (ref)	1.10(0.67-1.81)	0.84(0.47-1.51)	
TG+TT	179/416	220/409	202/420	0.78(0.51-1.20)	1.03(0.67-1.58)	0.83(0.51-1.36)	0.21	213/400	213/410	175/435	0.74(0.49-1.10)	0.74(0.48-1.14)	0.62(0.36-1.07)	0.89
	Fruit and vegetables							Alcohol						
GG	101/154	114/157	91/159	1.00 (ref)	1.18(0.72-1.93)	1.06(0.61-1.86)		103/160	95/151	108/159	1.00 (ref)	0.90(0.55-1.46)	1.07(0.65-1.74)	
TG+TT	224/397	199/414	178/434	0.80(0.54-1.20)	0.78(0.51-1.19)	0.72(0.44-1.19)	0.64	196/408	196/429	209/408	0.74(0.49-1.12)	0.67(0.44-1.01)	0.70(0.45-1.09)	0.81
TCF7L2 rs7903146	Red and processed meat							Fibre						
CC+TC	249/546	290/529	297/527	1.00 (ref)	1.24(0.92-1.69)	1.22(0.84-1.76)		295/522	297/522	244/558	1.00 (ref)	1.04(0.75-1.44)	0.87(0.54-1.39)	
TT	17/ 40	31/ 31	24/ 44	0.92(0.41-2.07)	2.29(1.08-4.86)	1.25(0.59-2.67)	0.20	27/ 40	21/ 36	24/ 39	1.17(0.58-2.39)	1.14(0.51-2.54)	1.24(0.56-2.71)	0.80
	Fruit and vegetables							Alcohol						
CC+TC	289/514	297/535	250/553	1.00 (ref)	1.09(0.80-1.48)	0.96(0.64-1.45)		279/529	271/541	286/532	1.00 (ref)	0.90(0.67-1.20)	0.95(0.69-1.31)	
TT	34/ 41	18/ 36	20/ 38	1.45(0.75-2.83)	1.04(0.46-2.38)	1.18(0.53-2.65)	0.59	22/ 41	21/ 38	29/ 36	0.97(0.45-2.09)	0.95(0.44-2.06)	1.64(0.79-3.41)	0.29
BCL2 rs2279115	Red and processed meat							Fibre						
AA	96/172	87/164	85/162	1.00 (ref)	1.00(0.61-1.64)	0.99(0.57-1.72)		93/151	99/155	76/192	1.00 (ref)	1.03(0.61-1.73)	0.69(0.38-1.29)	
CA+CC	163/405	221/390	226/402	0.70(0.46-1.05)	1.03(0.68-1.57)	0.99(0.62-1.57)	0.17	217/402	210/397	183/398	0.80(0.52-1.21)	0.84(0.53-1.32)	0.80(0.45-1.41)	0.21
	Fruit and vegetables							Alcohol						
AA	83/153	97/176	88/169	1.00 (ref)	1.09(0.66-1.80)	1.09(0.63-1.90)		89/154	92/175	87/169	1.00 (ref)	0.74(0.45-1.22)	0.81(0.48-1.35)	
CA+CC	231/391	205/393	174/413	1.02(0.67-1.56)	0.99(0.64-1.53)	0.85(0.51-1.40)	0.51	201/404	187/403	222/390	0.74(0.48-1.14)	0.69(0.45-1.06)	0.82(0.53-1.28)	0.37

N_c, N_{cases}; N_s, $N_{subcohort}$.
Number of missing cases; *SLC25A20* rs7623023 254, *PRKAB1* rs4213 257, *LPCAT1* rs7737692 262, *PLA2G4A* rs4402086 266, *ALOX5* rs3780894 259, *PTGER3* rs6685546 259, *TP53* rs1042522 266, *CCAT2* rs6983267 272, *TCF7L2* rs7903146 268, *BCL2* rs2279115 320.
[1]P-value for interaction
